# Foreign Body Response to Subcutaneous Implants in Diabetic Rats

**DOI:** 10.1371/journal.pone.0110945

**Published:** 2014-11-05

**Authors:** Teresa Oviedo Socarrás, Anilton C. Vasconcelos, Paula P. Campos, Nubia B. Pereira, Jessica P. C. Souza, Silvia P. Andrade

**Affiliations:** 1 Department of General Pathology, Institute of Biological Sciences, Federal University of Minas Gerais, Belo Horizonte, Minas Gerais, Brazil; 2 Department of Livestock Sciences, University of Córdoba, Montería, Córdoba, Colombia; 3 Department of Physiology and Biophysics, Institute of Biological Sciences, Federal University of Minas Gerais, Belo Horizonte, Minas Gerais, Brazil; Instituto de Engenharia Biomédica, University of Porto, Portugal

## Abstract

Implantation of synthetic matrices and biomedical devices in diabetic individuals has become a common procedure to repair and/or replace biological tissues. However, an adverse foreign body reaction that invariably occurs adjacent to implant devices impairing their function is poorly characterized in the diabetic environment. We investigated the influence of this condition on the abnormal tissue healing response in implants placed subcutaneously in normoglycemic and streptozotocin-induced diabetes in rats. In polyether-polyurethane sponge discs removed 10 days after implantation, the components of the fibrovascular tissue (angiogenesis, inflammation, fibrogenesis, and apoptosis) were assessed. Intra-implant levels of hemoglobin and vascular endothelial growth factor were not different after diabetes when compared with normoglycemic counterparts. However, there were a lower number of vessels in the fibrovascular tissue from diabetic rats when compared with vessel numbers in implants from non-diabetic animals. Overall, the inflammatory parameters (neutrophil accumulation - myeloperoxidase activity, tumor necrosis factor alpha, and monocyte chemotactic protein-1 levels and mast cell counting) increased in subcutaneous implants after diabetes induction. However, macrophage activation (N-acetyl-β-D-glucosaminidase activity) was lower in implants from diabetic rats when compared with those from normoglycemic animals. All fibrogenic markers (transforming growth factor beta 1 levels, collagen deposition, fibrous capsule thickness, and foreign body giant cells) decreased after diabetes, whereas apoptosis (TUNEL) increased. Our results showing that hyperglycemia down regulates the main features of the foreign body reaction induced by subcutaneous implants in rats may be relevant in understanding biomaterial integration and performance in diabetes.

## Introduction

The foreign body response refers to the non-specific immune response to implanted foreign materials [1]–[2]. It results from persistent inflammatory stimuli, such as the presence of implant devices in which a series of cellular alterations such as continuous inflammation are mediated by the various cell lineages. In this inflamed environment, macrophages, lymphocytes, mast cells, and their granular products contribute to the formation of foreign body giant cells (multinucleated fused macrophages) and the development of a dense of layer of fibrotic connective tissue which is detrimental to the implants’ function, safety, and biocompatibility [1]–[4]. In an experimental model of foreign body reaction induced by polyether-polyurethane sponge implants in normoglycemic animals, we have characterized a series of overlapping events, such as leukocyte/mast cell recruitment, angiogenesis, fibrogenesis, apoptosis, and the formation of foreign body giant cells [5]–[6]. All these features that vary in intensity and severity depending on the nature of the implanted material, characterize the host response to foreign materials in a number of different studies [4], [7]. Thus, while much is known about the foreign body reaction to indwelling medical devices in normoglycemic experimental animals, there is little information on the diabetic body’s response to injury inflicted by internal implants [8]. This is despite the fact that complications associated with the diabetes often require implantable devices such as glucose sensors, orthopedic implants, catheter, vascular grafts, drug-eluting stents, artificial organs, biosensors, scaffolds for tissue engineering, heart valves, and others [9]. In one study involving diabetic baboons bearing polystyrene subcutaneous implants, the inflammatory cell response, granulation tissue, and connective tissue ingrowth were shown to be reduced [10]. Similarly, delayed in matrix maturation and angiogenesis and higher numbers of inflammatory cells in titanium fiber mesh were seen around percutaneous implants in diabetic rabbits [11]. However, the prevalence of diabetes and the potential number of individuals that will need indwelling medical devices warrant further investigation to determine to what extent the diabetes state modifies the features of foreign body reaction. Therefore, we investigated the influence of hyperglycemia in the inflammatory cells recruitment/activation, neovascularization, apoptosis, foreign body giant cell, and fibrous capsule induced by synthetic matrix implanted subcutaneously in rats. We have shown that the pattern of the reaction to the synthetic polyether-polyurethane matrix differs in important ways between diabetic and non-diabetic rats.

## Materials and Methods

### Ethics Statement

The use of animals and procedures for this study was approved by the Ethics Committee of Animal Experimentation (CETEA) of Federal University of Minas Gerais, (protocol number 176/11). All surgery was performed under ketamine and xylazine anesthesia, and all efforts were made to minimize suffering.

### Animals

We used male Wistar laboratory rats weighing 300–350 g provided by Centro de Bioterismo (CEBIO) of Universidade Federal de Minas Gerais (UFMG). The animals were housed in polypropylene cages inside a well-ventilated room, provided with chow pellets and water *ad libitum* and maintained under a 12-hour light/dark cycle. All animal procedures were in accordance with the standards set forth in the guidelines for the care and use of experimental animals by our local Institutional Animal Welfare Committee.

### Induction of Diabetes Mellitus

Streptozotocin (STZ) was obtained from Sigma – Aldrich, St. Louis, MO, USA. STZ was dissolved in 10 mM citrate buffer (pH 4.5) and always prepared for immediate use within 5 to 10 min. STZ doses were determined according to the animals’ body weight and were administered intravenously in a single 60-mg/kg injection. The blood glucose level was measured before injection and subsequently every 4 days with On Call Plus Blood Glucose Meter (ACON Laboratories, Inc. San Diego CA, USA). Animals whose blood glucose level exceeded 200 mg/dl after treatment were considered diabetic.

### Preparation of sponge discs and implantation

Polyether–polyurethane sponge discs, 5 mm thick×12 mm diameter (Vitafoam Ltd, Manchester, U.K.) were used as the matrix for fibrovascular tissue growth. The sponge discs were soaked overnight in 70% ethanol and sterilized by boiling in distilled water for 30 min prior to the implantation surgery.

Thirteen days after STZ injection in the diabetic groups, all animals were anesthetized with a mixture of ketamine and xylazine (60 mg/kg and 10 mg/kg, respectively). The dorsal hair was shaved and the exposed skin wiped with 70% ethanol. Two sponges discs per animal (11 non diabetic and 12 diabetic) were aseptically implanted into a subcutaneous pouch through a 1 cm long dorsal mid-line incision. The incisions were closed with a silk braided nonabsorbable suture.

At 10 days post implantation (23 days after diabetes induction), the animals were anesthetized with ketamine and xylazine and later killed by cervical dislocation. The sponge discs were carefully dissected from the adherent tissue, removed, and weighed. They were then processed as described below and subjected to several assays.

### Tissue extraction and hemoglobin (Hb) measurement

Sponge implant vascularization was evaluated indirectly by the amount of Hb in the tissue detected by the Drabkin method. This method has been modified and used to determine angiogenesis in various experimental models and tissues, including the sponge implant model [6], [12]–[13]. All implants were individually homogenized (Tekmar TR-10, Cincinnati, OH) in 5 mL of Drabkin reagent (Labtest, São Paulo, Brazil) and centrifuged at 12.000 rpm for 20 min. The supernatants were filtered through a 0.22 mm filter (Millipore, São Paulo, Brazil). Hb concentration in the samples was determined spectrophotometrically by measuring absorbance at 540 nm using an enzyme linked immunosorbent assay (ELISA) plate reader and compared against a standard Hb curve. The Hb content in the implant sponge was expressed as µg Hb/mg of wet tissue.

### Determination of myeloperoxidase (MPO) activity

Neutrophil infiltration in the implants was measured indirectly by assaying MPO activity as previously described [14]. After using the sponge implant for Hb measurement, a part of the pellet was weighed and homogenized in 2 mL of pH 4.7 phosphate buffer (0.1 M NaCl, 0.02 M Na_3_PO_4_, 0.015 M NaEDTA, pH 4.7), and centrifuged at 12,000×g for 15 min. The pellet was resuspended in 0.05 M sodium phosphate buffer (pH 5.4) containing 0.5% hexa-1,6-bisdecyltrimethylammonium bromide (HTAB, Sigma Chemical Co., USA). Afterwards, the suspensions were freeze–thawed three times using liquid nitrogen and centrifuged at 10,000×g for 10 min. MPO activity in the supernatant samples was evaluated by quantifying the change in absorbance (optical density, OD) at 450 nm using tetramethylbenzidine (1.6 mM) and H_2_O_2_ (0.3 mM). The reaction was ended by adding 50 µl of H_2_SO_4_ (4 M). Results were expressed as a change in OD/mg of wet tissue.

### Determination of N-acetyl-β-D-glucosaminidase (NAG) activity

The extent of mononuclear cells in the sponge implants was quantitated by measuring the levels of the lysosomal enzyme, N-acetyl-glucosaminidase (NAG), present in high levels in activated macrophages [12], [15]–[16]. The implants were homogenized in 2 mL NaCl solution (0.9% w/v) containing 0.1% v/v Triton X-100 (Promega, Madison, WI) and centrifuged at 3000 rpm; 10 min at 4°C. One hundred µl of the supernatant was incubated for 10 min with 100 mL of pnitrophenyl-N-acetyl-b-D-glucosaminide (Sigma, Saint Louis, MO) prepared in citrate/phosphate buffer (0.1 M citric acid, 0.1 M Na_2_HPO_4_; pH 4.5) with a final concentration of 2.24 mM. The reaction was terminated by the adding 100 µl of 0.2 M glycine buffer (pH 10.6). Hydrolysis of the substrate was determined by the color absorption at 400 nm. A standard curve was constructed with *p*-nitrophenol (0–500 nmol/ml) and NAG activity was expressed as a change in nmol/mg of wet tissue.

### Measurement of VEGF, TNF-α, MCP-1, and TGF- β1 content of the sponge implants

Implants were homogenized in PBS pH 7.4 containing 0.05% Tween and centrifuged at 10,000×*g* for 30 min. The cytokines vascular endothelial growth factor (VEGF), tumor necrosis factor alpha (TNF-α), monocyte chemotactic protein-1 (MCP-1), and Transforming growth factor beta (TGF- β1) were measured in 100 µl of the supernatant using Immunoassay Kits (R and D Systems, USA) and following the manufacturer’s protocol. Dilutions of cell-free supernatants were added to ELISA plates coated with a specific murine monoclonal antibody against cytokine, followed by adding a second horseradish peroxidase-conjugated polyclonal antibody against cytokine. After washing to remove any unbound antibody-enzyme reagent, a substrate solution (50 µL of a 1∶1 solution of hydrogen peroxide and tetramethylbenzidine 10 mg/ml in DMSO) was added to the wells. Color development was stopped after 20 min incubation with 2N sulphuric acid (50 µL) and color intensity was measured at 540 nm on a spectrophotometer (E max – Molecular Devices). Standards were 0.5-log10 dilutions of recombinant murine cytokines from 7.5 pg/ml to 1000 pg/ml (100 µl). The results were expressed as picogram of cytokine/mg of wet tissue.

### Histological staining, immunohistochemistry, and morphometric analysis

The sponge implants from both groups (non-diabetic and diabetic) were fixed in 10% buffered formalin, pH 7.4, and processed for paraffin embedding. Sections with a thickness of 5 mm were stained with hematoxylin/eosin (H&E) and Dominici for light microscopic studies. Picrossirius-red staining followed by polarized-light microscopy was used to visualize and identify collagen fibers. The presence of apoptosis was investigated by TUNEL (TdT mediated dUTP nick end labeling) in histological sections, with a thickness pf 5 µm, using a commercial kit (TdT-FragEL DNA Fragmentation Detection Kit, Cat QIA33; Calbiochem, San Diego, CA, USA) to identify cells in apoptosis in marked terminal fragments of DNA (portion 3′-OH), associated with the characteristically fragmented nuclear DNA, allowing the morphological identification of cells undergoing apoptosis. The method was applied according to the manufacturer's instructions.

Immunohistochemistry (IHC) reactions for the detection of endothelial cells/blood vessels were performed using the monoclonal antibody clone CD 31, (Fitzgerald MA, USA). Tissue sections (5 µm) were dewaxed and antigens retrieved from the citrate buffer (pH 6). The slides were boiled in the citrate buffer for 25 minutes at 95°C and then cooled for 1 hour in the same buffer. Sections were incubated for 5 min in 3% hydrogen peroxide to quench endogenous tissue peroxidase. Nonspecific binding was blocked by using normal goat serum for 10 minutes (1∶10 in phosphate-buffered saline) with 1% bovine serum albumin (in phosphate-buffered saline). The sections were then immunostained with monoclonal antibody to CD31 (1∶40 dilution, DAKO Corporation, Carpinteria, CA, USA) for 60 min at room temperature. After washing in Tris–HCl buffer, sections were incubated for 30 min at room temperature with byotinylated Link Universal Streptavidin- HRP (Dako; Carpinteria, CA, USA). The reactions were revealed by applying 3,3′-diaminobenzidine in chromogen solution (DAB) (Dako; Carpinteria, CA, USA). The sections were counterstained with hematoxylin and mounted in Permount (Fisher Scientific; NJ, USA). Immunostaining was performed manually, and the spleen was used as a positive control. Negative controls were carried out by omitting the primary antibody, resulting in no detectable staining. The expression of these proteins was evaluated on the basis of the extent of cytoplasmic immunolabeling in endothelial cells forming lumen in six high-power fields, regardless of staining intensity (×400).

The number of blood vessels and giant cells, area of total collagen, capsule thickness, and number of mast cells and apoptotic cells were evaluated morphometrically. To perform the morphometric analysis, images of sequential cross sections from each implant were obtained from 25 fields for total number of cells, 35 fields for the number of blood vessels, and 20 fields for the number of mast cells, apoptotic cells, and giant cells. The images were captured with a planapochromatic objective (40x) in light microscopy (final magnification = 400x). For collagen analysis and wall thickness, images were obtained from three representative fields at 20x (final magnification = 200x). The images were digitized through a JVC TK-1270/JCB microcamera and transferred to an analyzer (software Image-Pro Plus 4.5, Media Cybernetics, Inc. Silver Spring, MD, USA). A countable vessel was defined as a structure with a lumen whether or not it contained red blood cells.

### Statistics

Results are presented as mean±SEM. The assumptions of normality and homoscedasticity were determined for subsequent statistical analysis. Comparisons between the two groups (non-diabetic and diabetic groups) were made using Student’s t-test or Mann-Whitney for unpaired groups. P<0.05 was considered significant. Analysis of the data was carried out using the GraphPad Prism program 6.0 version (La Jolla, CA, USA).

## Results

A single intravenous injection of streptozotocin (STZ) (60 mg/kg) rendered the rats diabetic with blood glucose levels at 425.8±12 mg/dl five days after the treatment, which remained unaltered for the entire experimental period (23 days). The body weight of diabetic animals was affected by the diabetogenic treatment. At the beginning of the experiment, the mean weight of the animals was 229±4.4 g. Twenty-three days after the diabetogenic treatment, the animals weighed 267±8.3 g and the control animals gained 96.3 g (325±7) (Table 1). Macroscopically, no signs of rejection were observed at the implantation site in diabetic and non-diabetic rats.

**Table 1:** Blood glucose level and animal weight in non-diabetic and diabetic rats during the study.

		Non-diabetic (n = 11)	Diabetic (n = 12)	ANOVA
**Blood glucose level** **(mg/dL)** (mean ± SEM)	0th day	86.64±3.4	78.67±1.7	-
	23th days	99.45±3.4	462.6±22.1	<0,0001
**Animal weight (g)** (mean ± SEM)	0th day	219.8±6.5	237.7±4.9	-
	23th days	325.4±7.2	267.3±8.3	<0,0001

### Histological examination of sponge implants

**Figure 1:**
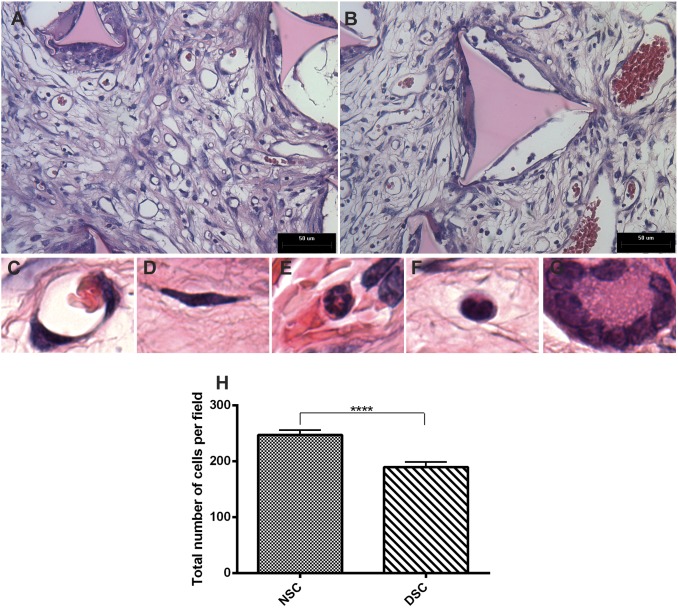
Histological analysis of 10-day old implants from non-diabetic and diabetic rats. Representative histological sections stained with H&E of fibrovascular tissue from diabetic (A) and non diabetic (B) rats. The pores of the sponge matrix, seen as triangular shapes, is composed of microvessels (C), spindle-shaped fibroblasts (D) and inflammatory infiltrate consisting of neutrophils (E), macrophages (F), and foreign body giant cells (G). Dilated microvessels and low cellularity were characteristics of implants from diabetic rats (H). Data are expressed as means ± SEM. *Significant difference between non-diabetic and diabetic; p<0.05; Mann-Whitney test. NSC, non-diabetic implant; DSC, diabetic implant; scale bar, 50 µm.

In histological sections of the implants (H&E), the synthetic matrix induced the formation of a fibrovascular tissue that differed in important ways between implants from diabetic and non-diabetic rats. In normoglycemic rats, the granulation tissue that filled the subcutaneous matrix was composed of spindle-shaped fibroblasts, microvessels, and a dense inflammatory infiltrate containing macrophages, neutrophils, and foreign body giant cells embedded in an extracellular matrix. However, in implants from diabetic rats, the connective tissue was immature with little matrix deposition. A lower number of blood vessels and cellularity was observed when compared with implants from non-diabetic animals. One striking difference between both implants was the vasodilation observed in the microvasculature in the hyperglycemic environment when compared with that in normoglycemic rats (Fig. 1A–H).

### Measurement of angiogenesis

**Figure 2:**
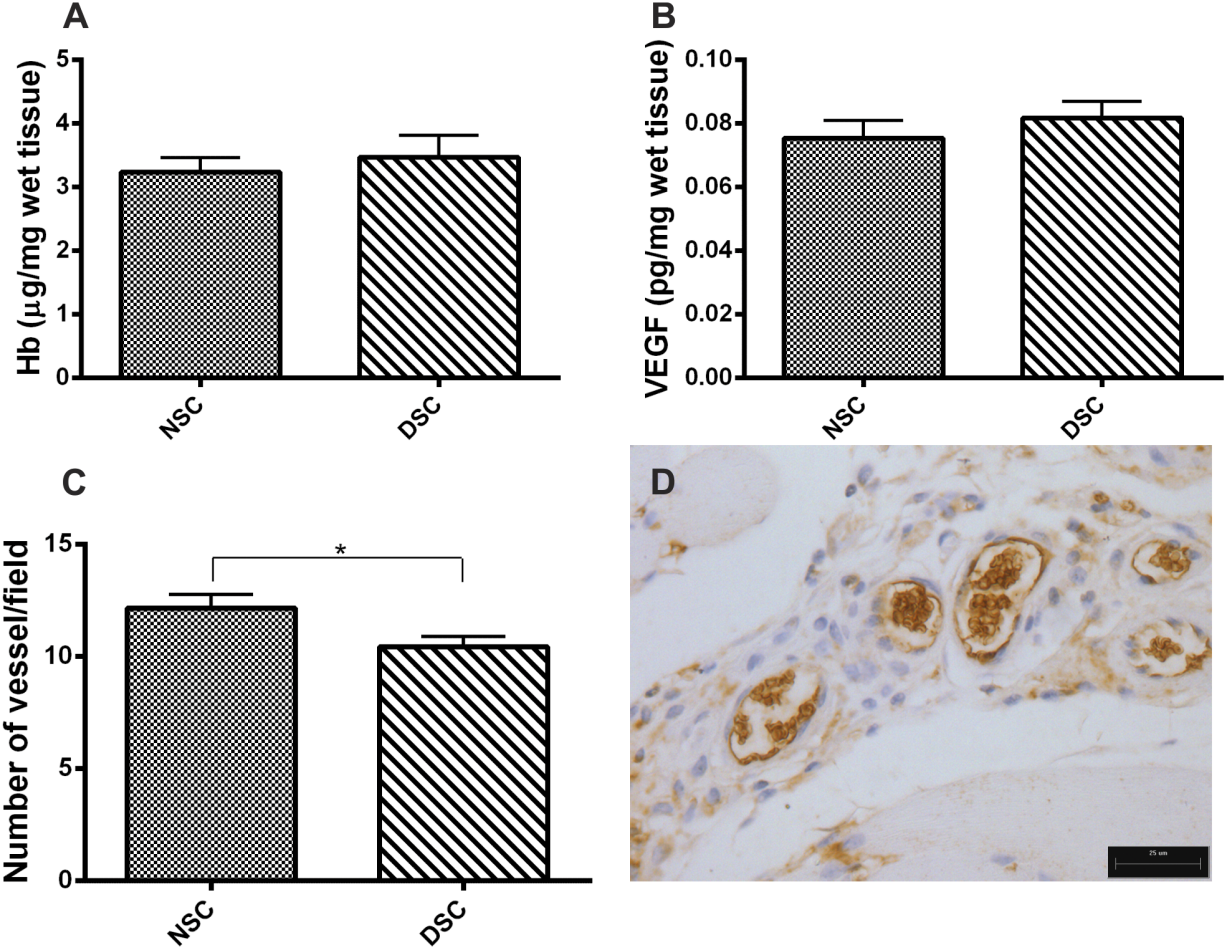
Vascularization in 10-day old implants from non-diabetic and diabetic rats. Hemoglobin content (A) and VEGF levels (B), both angiogenic markers, were not altered after diabetes induction. Morphometric analysis showed a decreased number of blood vessels in implants from diabetic as compared with non-diabetic rats (C). Representative CD31-immunostained section shows the newly formed vascular structures (D). Values shown are expressed as mean±SEM. *Significant difference between non-diabetic and diabetic; p<0.05. Student's t-test. NSC, non-diabetic implant; DSC, diabetic implant; scale bar, 25 µm.

The diabetogenic treatment did not alter the amount of Hb intraimplant or the levels of VEGF (a potent proangiogenic factor). However, the number of vessels, as determined in H&E stained sections and confirmed by CD31- immunostained sections, were lower in implants from diabetic rats (Fig. 2A–D).

### Inflammation in sponge implants

**Figure 3:**
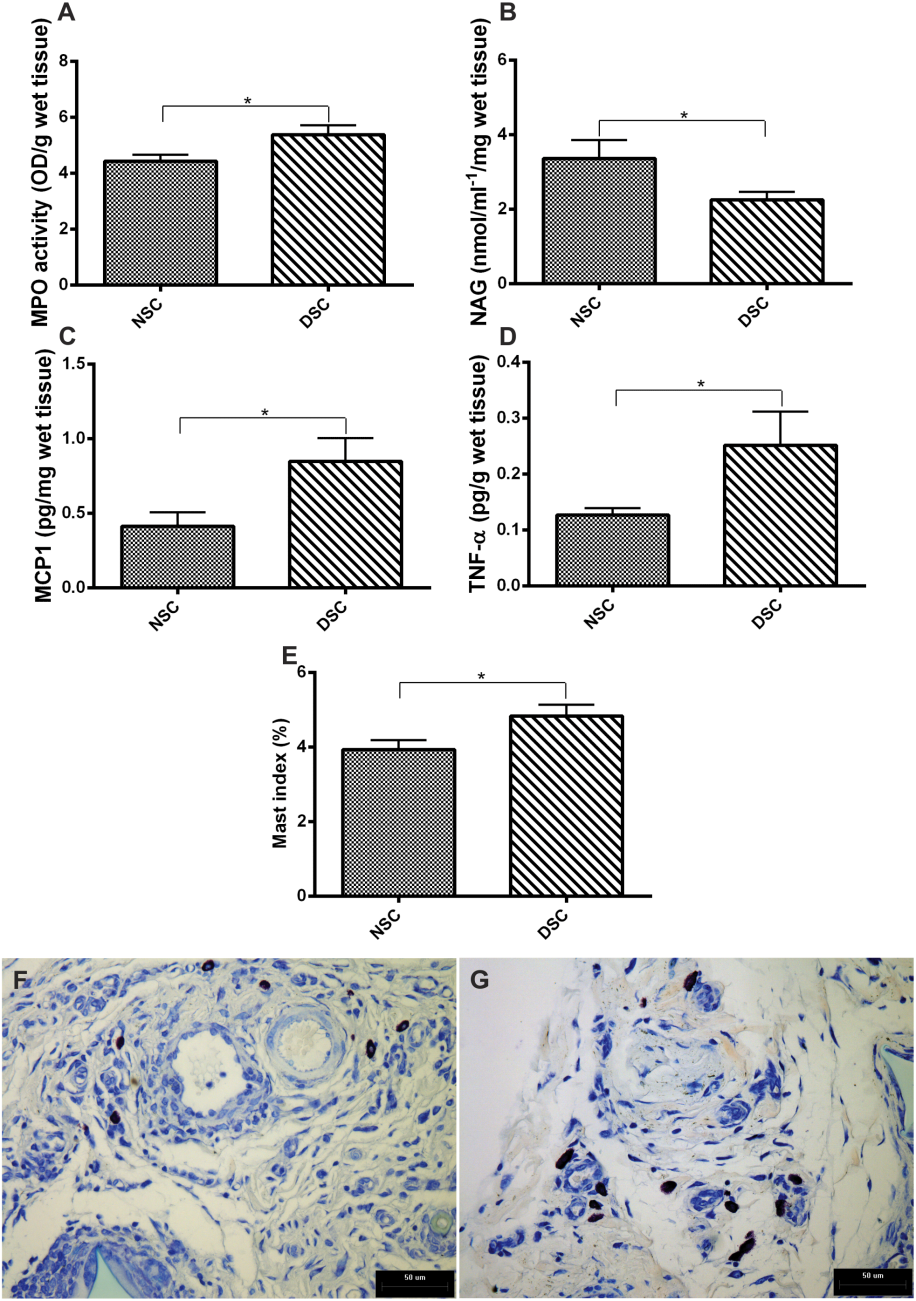
Markers of inflammation in 10-day old implants from non-diabetic and diabetic rats. Neutrophils and macrophages recruited to the implant were determined through MPO and NAG activities, respectively. An increase in MPO activity (A) and decreased in NAG activity (B) were observed after diabetes induction, indicating a persistence of acute inflammatory response and delay in the chronic response. An increase in other inflammatory indicators MCP-1 (C), TNF-α (D), and mast cell index (E) was observed. Representative histological images stained with Dominicci show mast cells in implants from non-diabetic (F) and diabetic rats (G). Values shown are expressed as mean±SEM. *Significant difference between non-diabetic and diabetic; p<0.05. Student's t-test and Mann-Whitney test. NSC, non-diabetic implant; DSC, diabetic implant; scale bar, 50 µm.

Several measurements of the inflammatory component of the implants (inflammatory enzyme activities and pro-inflammatory cytokines and mast cell index) were made. As shown in Fig. 3A–D, there were diabetes-related differences in leukocyte recruitment/activation in these parameters in 10-day old implants. In subcutaneous implants from non-diabetic rats, MPO activity, TNF-α, and MCP-1 levels were lower when compared with the values in implants from diabetic animals. NAG activity was higher in implants from non-diabetic animals than in diabetic rats. An increased number of mast cells (Dominici staining) was detected in implants from diabetic rats when compared with the number in implants from normoglycemic animals (Fig. 3E). Representative photomicrographs of mast cells in both groups are shown in Fig. 3F and 3G.

### Measurement of TGFβ1 levels and total collagen deposition

**Figure 4:**
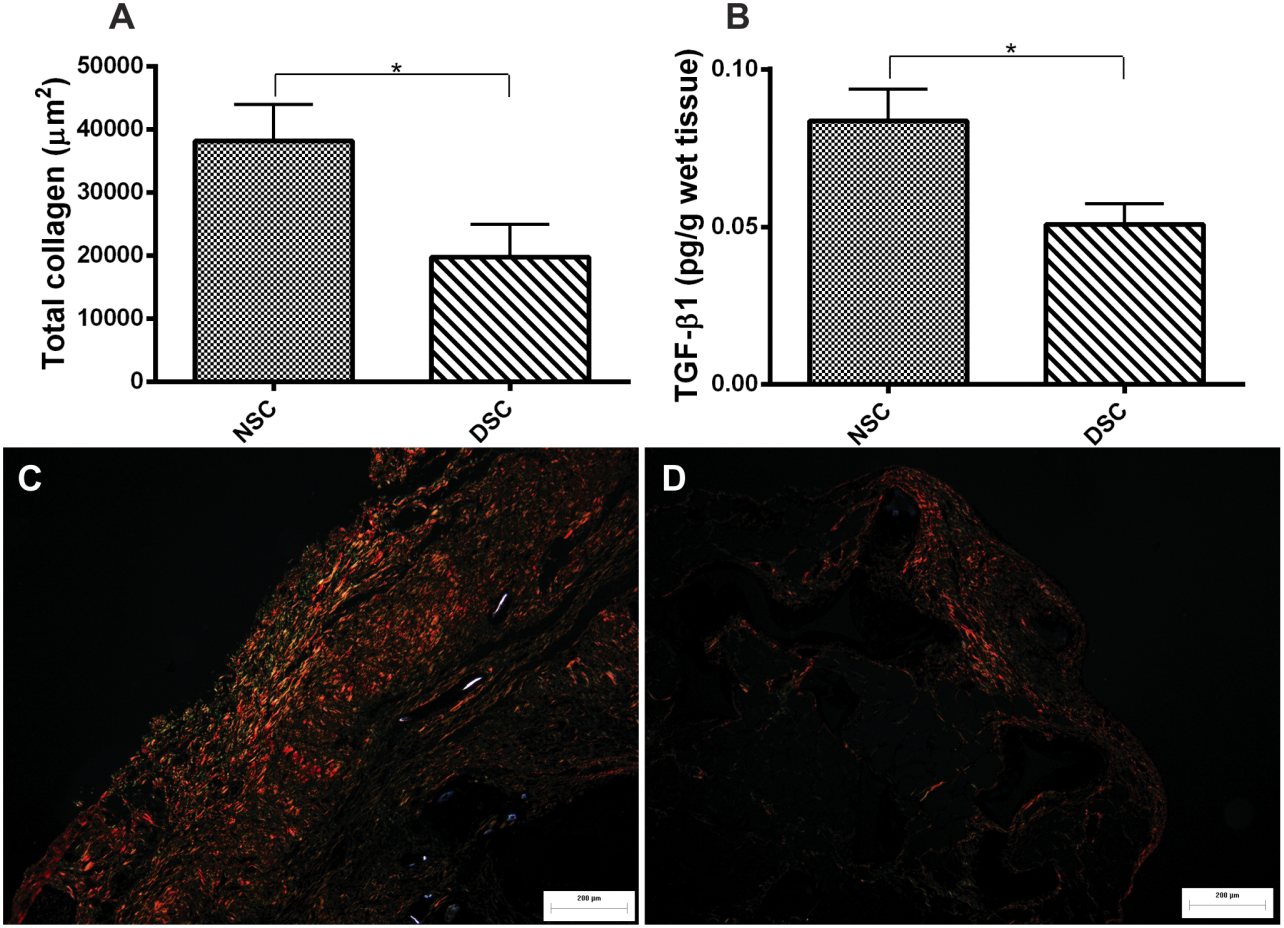
Markers of fibrogenesis in 10-day old implants from non-diabetic and diabetic rats. The amount of collagen (A) and levels of TGFβ1 (B) were increased in implants of non-diabetic rats as compared with that of diabetic rats. Representative histological sections (Picrossiurus-red staining) of implants from both groups of animals show distinct types of collagen in the implants (C) and (D). Values shown are expressed as mean±SEM. *Significant difference between non-diabetic and diabetic; p<0.05. Student's t-test. NSC, non-diabetic implant; DSC, diabetic implant; scale bar, 200 µm.

Fibrogenesis as an important wound healing and repair mechanism was measured by the cytokine TGF-β1 levels and total area of collagen (µm^2^) in the sponge implants (Picrossirius staining). A significant decrease in both parameters was observed in implants from diabetic when compared with that from non-diabetic rats (Fig. 4A–D).

### Apoptosis in sponge implants

**Figure 5:**
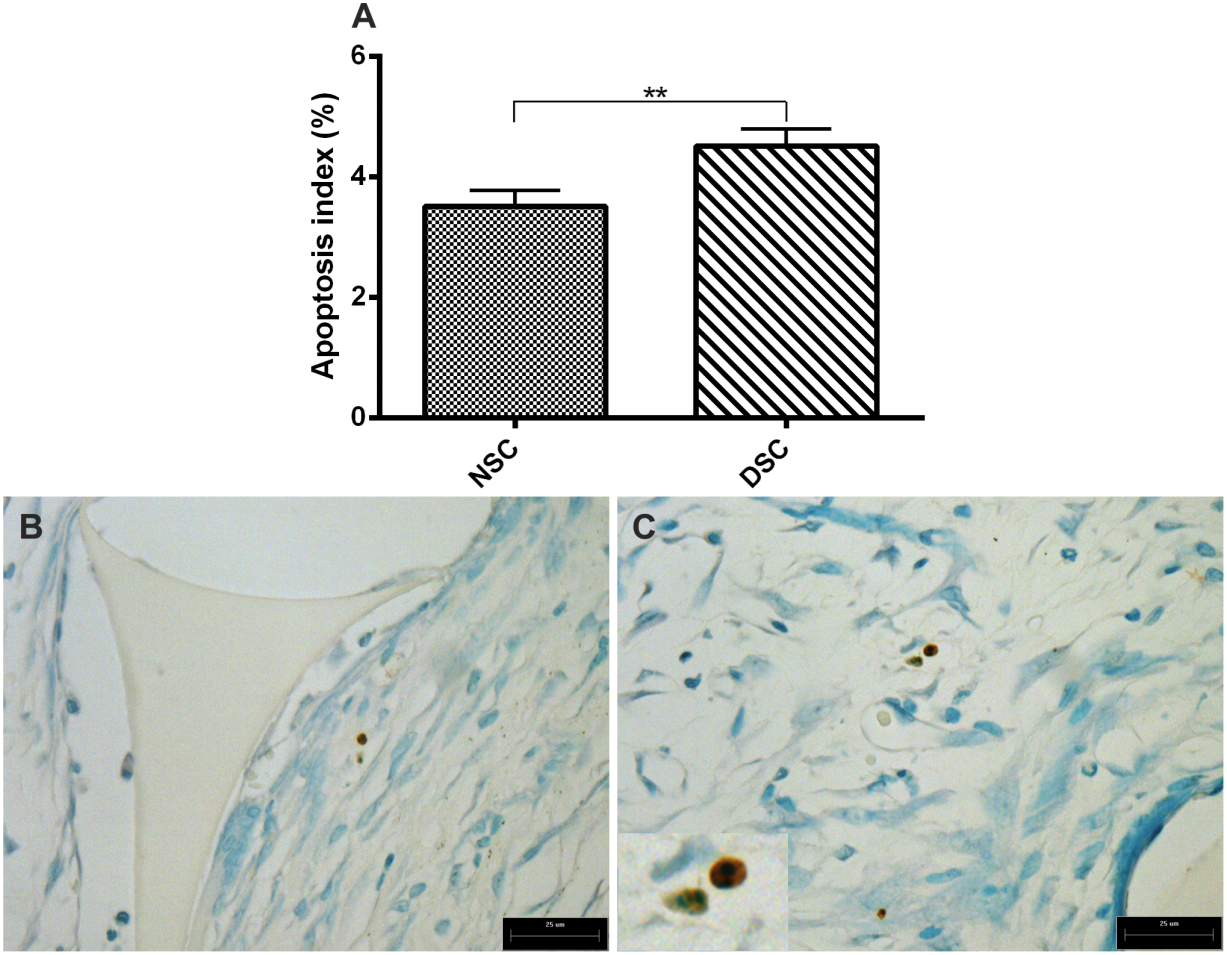
Pattern of apoptosis in 10-day old implants from non-diabetic and diabetic rats. Apoptotic index was increased in implants from diabetic rats as compared with non-diabetic rats (A). Values shown are expressed as mean±SEM. *Significant difference between non-diabetic and diabetic rats; p<0.05. Mann-Whitney test. NSC, non-diabetic implant; DSC, diabetic implant; scale bar, 25 µm. Representative histological sections (TUNEL staining) of the fibrovascular tissue induced by sponge implants at 10 post implantation show apoptotic cells in non-diabetic (B) and diabetic rats (C).

In sponge sections stained with TUNEL, the number of positive cells was clearly higher in implants from diabetic rats than it was in normoglycemic rats (Fig. 5A). Representative photomicrograph of apoptotic cells are presented in Fig. 5B and 5C. Dark-brown TUNEL positive nuclei with other morphological features of cellular death (apoptotic bodies, cellular shrinkage, and condensed chromatin) are clearly marked.

### Histological examination of the implant fibrous capsule and foreign body giant cells

**Figure 6:**
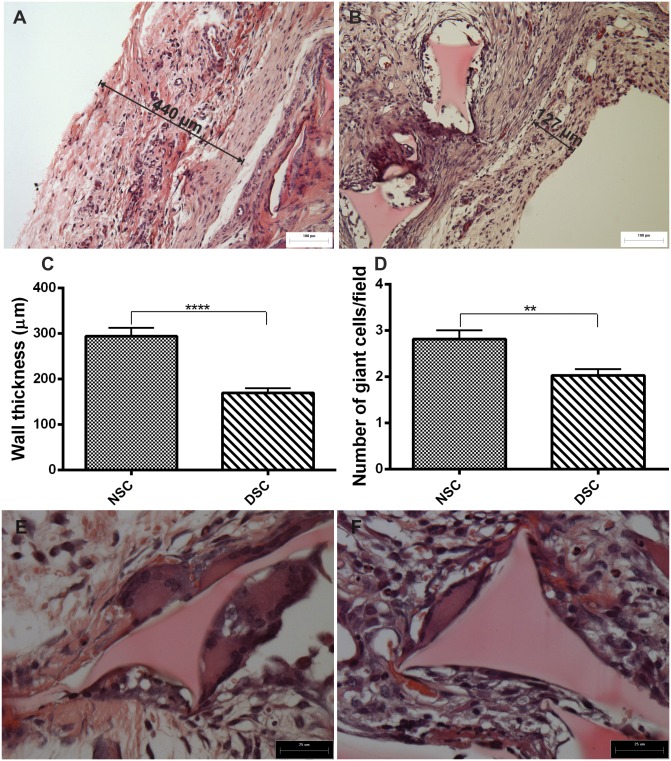
Histological characteristics of implant fibrous capsule. Representative histological sections of fibrous capsule in 10-day old implants from non-diabetic (A) and diabetic rats (B) (H&E staining) scale bar, 100 µm. Wall thickness was decreased after diabetes induction (C). Values shown are expressed as mean±SEM. *Significant difference between non-diabetic and diabetic; p<0.05. Student's t-test. Furthermore, the number foreign body giant cells was equally reduced in implants from diabetic rats (D). Values shown are expressed as mean±SEM. *Significant difference between non-diabetic and diabetic; p<0.05. Mann-Whitney test. Representative histological sections of foreign body giant cells in 10-day old implants from non-diabetic (E) and diabetic rats (F) (H&E staining); scale bar, 25 µm. NSC, non-diabetic implant; DSC, diabetic implant.

One of the most striking differences between the implants from normoglycemic and hyperglycemic animals was the thickness of the capsule. In implants from non-diabetic rats, the thickness was 294.5±18.5 µm versus 169.4±10.8 µm in implants from diabetic animals (Fig. 6A–C). Similarly, a decrease in the number of foreign body giant cells in implants from diabetic animals was observed when compared with those from non-diabetic animals (Fig. 6D–F).

## Discussion

Implantation of devices or biomaterials triggers a series of host reactions at the injury site that include interactions material/tissue, provisional matrix formation, acute and chronic inflammation, granulation tissue development, foreign body reaction, and fibrosis and fibrous capsule development [2], [7]. Invariably, this foreign body reaction affects the implanted material’s efficiency, leading to device failure [4], [8], [17]. Much of the knowledge on this adverse healing following biomaterial implantation has come from normoglycemic individuals, despite the fact that complications associated with diabetes often requires implantable devices such as glucose sensors, orthopedic implants, catheter, vascular grafts, drug-eluting stents, artificial organs, biosensors, scaffolds for tissue engineering, and heart valves in diabetic individuals [9]. By studying the inflammatory, angiogenic, apoptotic, and fibrogenic components of the foreign body response induced by subcutaneous polyether-polyurethane implants in rats, we have been able to identify a number of diabetes-related alterations of this reaction within and around the synthetic matrix.

In the descriptive histologic analysis of the implants from diabetic animals, a less mature and less dense extracellular matrix with fewer, but more dilated blood vessels (H&E stained sections and in CD31-immunostained sections) was observed in contrast with implants from non-diabetic rats. This result is consistent with reduced amounts of collagen and impaired angiogenesis in cutaneous wound healing and soft-tissue around implanted devices in diabetes [10]–[11], [18]–[19]. However, Hb content and VEGF levels (angiogenic markers) in implants from diabetic rats were not altered in our experiments. This apparent discrepancy between a decreased number of vessels in implants from diabetic animals and no change in hemoglobin content may be explained by the increased vasodilatation observed in the histological sections of implants from the same group. Vascular dysfunctions, such as increased permeability, vasodilatation, and structural changes, are well established abnormalities in the diabetic state in experimental animal and human microvasculature [10], [20]–[21]. Because we have shown an increased number of mast cells in implants from diabetic animals when compared with that from normoglycemic rats, it is possible that the histamine’s well-known vasodilator effect may have contributed to maintaining the Hb content in both implants, despite the smaller number of blood vessels in implants from diabetic rats.

The inflammatory components of the skin wound’s microenvironment in diabetes is altered in several aspects, including abnormal cellular infiltration, cytokine production, and protease release [2], [22]–[23]. However, less is known about the inflammatory component of internal injury following device implantation in diabetes [8]. We measured four inflammation markers (MPO and NAG activities and two cytokines, TNF-α and MCP-1levels), in 10-day old implants from both groups of animals. We have also determined the number of mast cells within the fibrovascular tissue in the implants. All parameters, except for NAG activity (macrophage activation), were higher in implants from diabetic rats when compared with those from non-diabetic animals. We did not expect to find that, in implants from diabetic rats, the levels of MCP-1 (relevant chemoattractant chemokine) did not parallel NAG activity as previously demonstrated [24]. One possible explanation for this discrepancy is that, in the hyperglycemic environment, macrophages may be defective in their response to the chemoattractant stimulus. Alternatively, MCP-1 production and macrophage recruitment in the foreign body reaction may differ between internal and skin injuries. Apart from this, our findings are in accordance with the notion of an altered inflammatory microenvironment in diabetic skin wounds and this concept is extended to the foreign body reaction in rats. To same extent, our results are also in agreement with the studies performed in subcutaneous and percutaneous implants from diabetic baboons and rabbits, in which persistent infiltration of neutrophils and a reduced number of macrophages were observed in internal wounds [10]–[11]. These changes have occurred together with changes in chemokine and growth factor expression (increase levels of MCP-1 and TNF-α). An increased production of the same inflammatory cytokines was observed in the wounds of type-1 diabetic patients [25]. In the excisional skin wound mice model of type 1 and 2 diabetes, increased TNF-α levels were reported and proposed to account for fibroblast apoptosis, which, in turn, resulted in a failure to produce sufficient matrix deposition at the injury site [26]. In our findings, TNF-α levels and the apoptotic index were also higher in the internal fibrovascular tissue in diabetes. These may be one possible explanation for the decreased levels of TGB-β1 production, collagen deposition, fibrous capsule formation and number of foreign body giant cells found in implants from diabetic rats compared with that from normoglycemic animals. In fact, it has been proposed that processes that interfere with sufficient number of cells involved in the various phases of repair, such as fibroblast apoptosis, impair wound healing [26]–[28].

Numerous reports indicate that encapsulation is influenced by several factors, including properties of the biomaterial, biomaterial porosity, surface texture, and implantation site [29]. In fact, our observation of the events following intraperitoneal (i.p.) implantation of sponge discs in diabetic rats showed increased levels of angiogenic (VEGF) and fibrogenic (TGF-β1) cytokines and an increase in macrophage accumulation [30] which are in contrast with the findings from subcutaneous implants in the hyperglycemic environment. Nevertheless, the outcome of our experiments was an attenuated fibrogenic response in both intraperitoneal and subcutaneous implants in diabetic animals.

The main findings that have emerged from our work were that the changes observed in the foreign body reaction induced by synthetic implants in diabetic rats shared similarities with those seen in cutaneous tissue reported in the literature (decreased collagen deposition, disturbed inflammatory infiltrate, decreased angiogenesis, and increased apoptosis) in diabetic animals. More importantly, fibrous capsule formation and presence of foreign body giant cells, the typical features of the foreign body reaction, were attenuated in the hyperglycemic environment. These findings may be critical in developing strategies that may improve the performance and function of implanted biomaterials in diabetes.

## Supporting Information

Data S1 **Analyses of implants from normoglycemic and hyperglycemic animals.**
(PDF)
