# Supplementary material for: Foreign Body Response to Subcutaneous Implants in Diabetic Rats
Source: PLoS One. 2014 Nov 5;9(11):e110945. doi: 10.1371/journal.pone.0110945 (PMC4220951; doi:10.1371/journal.pone.0110945)
Supplement: Data S1 — Analyses of implants from normoglycemic and hyperglycemic animals. (PDF) [file pone.0110945.s001.pdf]

Days post-STZ	Non diabetic - glycaemia										
	**1**	**2**	**3**	**4**	**5**	**6**	**7**	**8**	**9**	**10**	**11**
**0**	75	75	70	70	102	103	89	88	91	88	92
**4**	95	95	103	92	107	93	116	109	100	111	100
**8**	96	96	99	95	108	92	97	85	105	100	107
**12**	99	99	95	93	106	93	113	106	88	106	107
**16**	101	101	105	89	96	83	92	79	99	89	92
**20**	103	103	108	92	89	71	96	97	89	85	78
**23**	109	109	99	86	104	78	108	97	104	118	93

Days post-STZ	Diabetic - glycaemia											
	**1**	**2**	**3**	**4**	**5**	**6**	**7**	**8**	**9**	**10**	**11**	**12**
**0**	78	88	82	70	72	75	87	73	76	79	81	83
**4**	501	458	472	484	373	553	528	428	389	467	500	380
**8**	568	497	431	407	273	438	130	371	351	561	554	391
**12**	466	380	470	393	386	402	320	362	397	374	540	328
**16**	472	425	400	462	318	449	341	262	354	403	476	362
**20**	421	345	371	468	320	447	417	372	428	488	507	463
**23**	558	377	412	363	362	519	425	445	475	529	583	503

Days post-STZ	Non diabetic - Animal weight (g)										
	**1**	**2**	**3**	**4**	**5**	**6**	**7**	**8**	**9**	**10**	**11**
**0**	238	211	208	256	218	192	208	203	251	234	199
**4**	269	249	233	290	247	231	257	241	294	270	244
**8**	294	278	265	324	279	256	279	274	321	301	270
**12**	310	295	284	341	298	266	278	293	335	293	265
**16**	313	307	295	340	288	276	287	305	342	292	283
**20**	313	307	295	340	288	276	287	305	342	292	283
**23**	326	330	321	363	305	285	305	340	365	319	320

Days post-STZ	Diabetic - Animal weight (g)											
	**1**	**2**	**3**	**4**	**5**	**6**	**7**	**8**	**9**	**10**	**11**	**12**
**0**	258	252	212	247	234	234	222	221	214	251	249	258
**4**	248	212	261	245	232	222	237	216	210	239	230	258
**8**	272	226	285	260	249	224	266	229	226	246	248	279
**12**	272	224	279	270	267	235	264	235	223	254	238	289
**16**	278	230	295	275	270	244	260	225	216	262	230	293
**20**	267	227	300	262	261	237	279	234	235	259	222	301
**23**	290	220	308	264	274	251	289	250	238	274	239	310

Hb (mg/mg wet tissue)	
**NSC**	**DSC**
3,7	3,6
3,7	2,7
3,3	3,9
3,0	4,5
3,3	3,6
4,2	4,0
2,5	3,1
4,3	1,4
2,3	5,5
1,9	4,0
3,4	1,5
-	3,9

VEGF (pg/mg wet tissue)	
**NSC**	**DSC**
0,07	0,06
0,07	0,06
0,06	0,08
0,07	0,07
0,06	0,09
0,12	0,07
0,07	0,08
0,08	0,07
0,07	0,11
0,06	0,09
0,10	0,12
-	0,08

MCP1 (pg/mg wet tissue)	
**NSC**	**DSC**
0,432	1,168
0,483	0,505
0,892	1,401
0,130	1,037
0,643	0,671
0,581	0,195
0,057	0,508
0,106	0,418
0,402	1,565
-	0,264
-	1,597

MPO activity (OD/g wet tissue)	
**NSC**	**DSC**
5,16	7,02
5,22	4,99
3,83	3,88
4,08	4,75
4,51	5,58
3,49	6,94
5,12	5,81
5,62	4,72
3,92	4,85
3,35	4,08
4,45	4,69
-	7,27

NAG (nmol/ml-1/mg wet tissue)	
**NSC**	**DSC**
2,46	3,84
2,12	2,45
4,30	3,22
1,82	1,72
3,60	2,29
1,81	1,60
6,12	1,95
6,27	2,02
2,72	2,40
1,86	1,57
3,91	1,71

TGF-b1 (pg/g wet tissue)	
**NSC**	**DSC**
0,10	0,02
0,11	0,05
0,06	0,05
0,14	0,06
0,04	0,02
0,06	0,07
0,11	0,07
0,05	0,08
0,12	0,03
0,07	0,07
0,06	0,07
-	0,02

TNF-a (pg/g wet tissue)	
**NSC**	**DSC**
0,09	0,20
0,09	0,16
0,18	0,52
0,14	0,14
0,11	0,16
0,14	0,33
0,14	-

Apoptosis index (%)	
**NSC**	**DSC**
1,89	3,10
3,66	7,50
2,02	9,10
1,32	3,70
2,13	4,90
1,75	5,30
2,56	5,30
1,54	2,50
1,75	3,30
0,00	2,00
1,33	2,10
1,74	3,20
1,67	5,90
4,23	2,00
1,89	2,00
2,35	*11,10**
2,53	7,10
4,17	5,60
1,69	3,60
2,70	5,60
4,11	9,50
8,20	*33,30**
1,56	2,00
1,64	3,20
5,26	3,30
4,00	*33,30**
2,22	2,60
7,14	6,30
6,52	3,40
2,86	3,00
6,06	6,80
8,33	8,30
3,13	5,30
2,50	3,60
5,41	4,00
4,23	2,80
2,38	8,30
3,70	7,69
3,08	3,57
5,56	4,76
4,44	4,00
2,17	4,17
4,76	3,57
2,33	3,57
6,67	2,78
4,76	2,08
3,17	4,17
5,63	2,94
5,00	2,50
5,26	7,89
1,96	3,70
5,88	2,78
3,70	4,17
	7,55
	2,94
	2,94
	3,03
	5,00
	3,45
	6,67
	10,00

Total collagen (μm^2)	
**NSC**	**DSC**
25930,47	6527,143
27960,11	1010,977
37424,08	1438,944
32057,40	8305,642
53473,71	9853,500
45847,61	19185,500
12133,00	34902,830
28593,09	25084,360
22921,03	15868,530
39419,13	11564,940
40301,97	57272,880
91964,72	46656,990

Wall thickness (mm)	
**NSC**	**DSC**
79,02	187,01
171,21	146,45
143,81	202,77
258,85	174,88
332,21	178,90
309,63	289,05
373,47	208,65
402,43	231,86
431,21	254,61
144,13	218,39
186,27	263,20
235,27	253,03
317,98	133,06
285,29	132,78
343,75	113,07
228,62	133,55
370,51	159,50
264,24	91,23
340,80	122,21
311,35	186,99
340,82	195,00
245,69	122,26
283,84	136,68
272,50	90,40
367,39	109,68
465,84	134,01
444,21	103,55

Number of giant cells/field	
**NSC**	**DSC**
2	2
3	2
2	1
1	1
1	2
4	1
2	2
1	3
2	1
1	2
3	2
4	5
2	1
3	2
1	2
1	2
5	1
5	3
2	4
3	3
5	2
4	2
4	1
4	1
2	1
1	2
3	3
3	1
2	1
3	4
3	1
2	2
4	1
2	2
5	3
2	1
1	2
4	3
4	3
3	2
4	1
1	1
2	1
1	2
2	4
2	2
4	2
3	2
1	1
3	1
3	2
9	4
4	3
3	2
4	1
3	5
1	3
2	1
4	2
4	2

Number of vessel/field	
**NSC**	**DSC**
21	12
19	12
17	9
13	9
14	11
16	13
4	14
13	19
20	6
11	17
13	10
11	11
13	19
14	9
13	8
14	13
4	14
19	11
10	16
13	19
6	16
12	8
13	9
7	15
27	15
25	17
26	9
9	6
10	15
18	11
6	8
11	15
8	10
4	10
13	3
6	12
10	11
5	8
5	5
3	4
7	2
7	5
4	7
9	9
11	4
5	7
19	8
12	4
9	8
8	7
8	9
18	3
26	9
12	10
19	10

13	3
4	13
6	6
8	7
10	13
7	16
10	11
7	6
10	10
11	17
7	8
6	8
11	11
13	12
5	11
24	13
17	8
23	2
15	8
9	11
13	14
6	16
2	19
11	12
20	12
10	7
21	11
19	6
20	13
5	19
15	14
12	11
11	13
9	8
8	
17	
28	
21	
20	
10	
13	
8	
12	
7	

Mast index per field	
NSC	DSC
4	12
5	5
5	6
7	8
13	10
4	8
6	6
5	10
5	8
15	6
6	6
8	8
11	4
19	12
9	15
13	11
15	4
6	5
8	3
4	7
10	5
23	2
14	5
5	9
8	6
4	8
7	9
3	9
4	9
15	14
7	8
7	14
5	8
9	9
4	9
16	6
9	7
21	9
22	7
6	2
2	10
11	11
8	6
9	12
4	7
21	5
8	5
18	5
9	13
19	9
7	21
3	15
4	12
11	8
7	11

17	5
20	5
11	9
7	5
12	4
12	2
8	3
6	8
14	10
17	7
13	15
15	12
7	9
11	13
5	11
3	8
	2
	10
	3
	4
	3
	7
	6
	2
	5
	6
	5
	7
	6
	18
	7
	9
	11
	5

Total number of cells per field	
**NSC**	**DSC**
370	214
270	486
278	112
342	132
244	148
226	128
264	189
263	266
225	136
216	281
204	197
173	301
184	157
208	424
213	373
245	450
246	316
226	73
184	415
197	107
311	229
365	128
302	140
310	118
225	126
106	217
168	121
240	42
205	185
103	172
141	207
213	252
227	228
224	220
240	183
147	163
229	187
377	177
313	162
437	142
147	182
140	162
159	168
337	202
402	184
305	152
360	176
361	432
361	236
291	191
298	274
252	160
227	199

211	203
358	164
190	113
241	176
313	269
183	172
170	281
231	132
249	120
237	99
193	135
341	171
226	214
264	271
205	221
243	201
297	111
96	152
	192
	112
	154
	65
	189
	128
	138
	94
	97
	118
	146
	188
	81
	166
	169
	248
	121
	252
